# In-vitro antibacterial activity and mechanism of *Monarda didyma* essential oils against *Carbapenem-resistant Klebsiella pneumoniae*

**DOI:** 10.1186/s12866-023-03015-4

**Published:** 2023-09-20

**Authors:** Ying Chen, Jinda Zhao, Chenyu Liu, Dongmei Wu, Xianhe Wang

**Affiliations:** 1 Key laboratory of Microecology-immune Regulatory Network and Related Diseases School of Basic Medicine, Jiamusi University, NO. 148 Xuefu Street, Xiangyang District, Jiamusi, 154000 Heilongjiang Province China; 2 The First Affiliated Hospital of Jiamusi University, NO 348 Dexiang street, Xiangyang district, Jiamusi, 154000 Heilongjiang Province China

**Keywords:** CRKP, MDEO, Antibacterial activity, Mechanisms, In vitro

## Abstract

To fight the global epidemic of drug-resistant bacteria, essential oils have gained increasing attention as a new source of antibiotics. The antimicrobial activity of *Monarda didyma* essential oils (MDEO) for the *Carbapenem-resistant Klebsiella pneumoniae* (CRKP) strains were determined by agar disc diffusion assay and broth microdilution assay. To further understand MDEO efficacy, a time-growth curve was performed. The biofilm formation of CRKP were determined by crystalline violet staining method, additionally, changes in intracellular Adenosine triphosphate (ATP), protein, Alkaline phosphatase (AKP) activities, and membrane integrity were investigated to assess the influence of MDEO on cell membrane damage. Finally, the activities of key enzymes in the tricarboxylic acid (TCA) pathways and pentose phosphate (PPP) pathways were examined to determine the effect of MDEO on the respiratory metabolism of CRKP. This study presents the antibacterial mechanism of MDEO against CRKP with a minimum inhibitory concentration (MIC) and minimum bactericidal concentration (MBC) of 1.25 mg/ml. To understand MDEO efficacy, a time-kill kinetics approach was performed. The bactericidal effect of MDEO was evident at 2 h compared to the control at its MIC and 2MIC. Surface electron microscopic and ATP assay studies provided evidence for the multi-target action of MDEO against CRKP. MDEO could inhibit CRKP biofilm formation. MDEO could also cause irreversible damage to the CRKP cell membrane, resulting in the leakage of biological macromolecules (protein, ATP) and the reduction of intracellular enzymes (AKP) activities. Finally, MDEO affected the pathways of respiratory metabolism, such as PPP and TCA pathways. MDEO could reduce the activity of key enzymes (Glucose-6-phosphate dehydrogenase, citrate synthase, isocitrate dehydrogenase, and α-ketoglutarate dehydrogenase) in the PPP and TCA pathways to exert its biological effects against CRKP. These results suggest MDEO can exert inhibitory effects on CRKP, and potential mechanisms of action including inhibition of biofilm formation, damage of cell membrane structure and inhibition of energy metabolism.

**Supplementary Information:**

The online version contains supplementary material available at 10.1186/s12866-023-03015-4.

## Introduction

The world is witnessing a growing epidemic of infections due to Antibiotic resistance (AMR) as a result of indiscriminate and inadequate infection prevention [1]. An estimated 4.95 million deaths were associated with bacterial AMR in 2019 [2], without action, AMR will rapidly spread - infecting and killing more people yearly. *Carbapenem-resistant Klebsiella pneumoniae* (CRKP), belonging to the Carbapenem-resistant Enterobacteriaceae (CRE) family, can produce carbapenemases, enzymes that render carbapenems, penicillin, and cephalosporins ineffective. For this reason, they have been termed “nightmare bacteria”—with only a few alternative antibiotics for their management. In the Centers for Disease Control and Prevention (CDC) report, CRKP was listed as an urgent worldwide threat [3].

We cannot rely on antibiotics alone to solve the increasingly prevalent resistance occurrences. No new classes of antibiotics have been approved to treat gram-negative infections in the last 50 years [4, 5]. Therefore, finding a new bactericide that can effectively eliminate Multiple drug resistance (MDR) in the environment, especially in a clinical setting, without endangering human health, has become an urgent need. Accordingly, essential oils (EO) as a new source of antibiotics have gained increased scientific interest. Numerous studies demonstrate a range of EO biological properties, such as antibacterial, antiviral, antioxidant, anticancer, anti-inflammatory, and immunomodulatory [6]. Essential oils are complex mixtures of odorous volatile organic compounds, generally regarded as safe, environmentally friendly, and non-toxic. Their antimicrobial properties are mainly a result of the effects of EOs and their constituents, such as monoterpenes, sequesters, and phenylpropanoids, on the cytoplasmic cell membrane [7]. *Monarda didyma* studied in this work is a wild plant originating from North America and grown as an agricultural plant in Northeast China. *Monarda didyma* has been utilized in traditional medicine, famous for its effects on digestive disorders. It has also demonstrated anthelmintic, hypnotic, diuretic, expectorant, purgative, and detoxifying effects [8, 9]. However, its antibacterial properties have not been extensively studied. The main objective of this study was to evaluate *Monarda didyma* essential oils (MDEO) antimicrobial capacity and mechanisms against CRKP. The EO’s mechanisms of action were tested by its inhibitory effects on biofilm formation, cell membrane integrity, and respiratory metabolism [10].

## Methods

### Bacterial strains and culture conditions

The laboratory of the First Affiliated Hospital of Jiamusi University provided 20 subcultures of CRKP without human genetic information. All samplings of humans were performed in accordance with the relevant ethical principles and guidelines. Ethical approval for human sampling was obtained from The First Affiliated Hospital of Jiamusi University (2022 − 1299). Strains were identified by Bruker MALDI Biotyper (Bruker, USA). The KP antibiotic susceptibility was tested using the VITEK 2 Compact system (bioMérieux, Warsaw, Poland). *K. pneumoniae* strains ATCC 700,703 and ATCC BAA-1706 were used as reference strains. All bacterial cultures were stored in a Luria-Bertani (LB) liquid medium containing 25% (v/v) glycerol (Merck) at − 80^°^C. Cultures were revived on Muller-Hinton (MH) plates as necessary. All strains were cultured in tryptic soy broth (TSB, Sigma, USA). The bacterial concentration was adjusted by counting the colony-forming units (CFU) and spectrophotometrically measuring the OD_600_.

### Essential oils

*Monarda didyma L.* essential oil (MDEO) was extracted by hydrogenated distillation and given as a gift by Prof. Dongmei Wu (Jiamusi University, China). MDEO composition was assessed by GC-MS in previous studies [11], and consisted mainly of thymol (63.8%), 2-lignocaine, β-cinnamene, pinene, terpene, cyclamen, terpinene, and δ-3-carene.EO was diluted by 1% DMSO and preserved in a brown glass bottle at 4℃.

### Agar disc diffusion assay

0.5 MCF of the overnight cultured CRKP was spread on MHA plates. A sterile paper plate infiltrated with EO (pure oil) was then placed on the center of the CRKP-coated MHA plates. A Ceftazidime-avibactam infiltrated sterile paper plate (CZA, Oxoid, England), as well as 0.9% Sodium Chloride (20 µl, Normal Saline, NS), and 1% DMSO (20 µl, Hushi, China) infiltrated sterile paper plates were used as the control groups, respectively. The cultures were incubated at 37 °C for 18 h. The inhibition zone diameter was subsequently measured. Each experiment was performed independently three times, and the results were expressed as mean ± standard deviation.

### Minimum inhibitory concentration (MIC) and minimum bactericidal concentration (MBC) assays

The MIC and MBC of MDEO for the CRKP strains were determined by broth microdilution assays according to the protocol of the Clinical and Laboratory Standard Institute [12]. A bacterial suspension of 0.5 McFarland turbidity standard was initially diluted 1000-fold with LB broth (LB, Oxoid). The bacterial suspension was then mixed with MDEO. Essential oil concentrations were prepared from 128 mg/ml to 0.125 mg/ml. An LB broth containing CRKP served as the negative control. The MIC value was defined as the lowest MDEO concentration that resulted in non-significant bacterial growth after overnight incubation. For MBC value determination, cultures without significant bacterial growth were subcultured on MH plates and incubated for 18 h at 37^°^C. MBC was defined as the lowest MDEO concentration that eliminated the inoculum growth when subcultured [13]. Each experiment was performed independently three times.

### The growth curve analyses

The MDEO antibacterial properties against CRKP were analyzed by a growth-curve assay as previously described [14]. A 0.5 McFarland bacterial suspension was prepared in PSB, supplemented with serial dilutions of the EOs, with concentrations ranging from 2 to 1/16MIC, in 96-well plates. An equal volume of TBS containing 1% DMSO was used as a negative control. The samples were then cultured in an oscillating incubator at 37 °C and 150 r/min. The optical density at 600 nm was measured at 1-hour intervals using a Microplate-Reader (Biotek Synergy H1/Synergy2, USA). The growth curve was plotted using OD_600_ as the vertical coordinate and incubation time as the horizontal coordinate. Each experiment was performed independently three times.

### Scanning electron microscope (SEM)

SEM assays were carried out to determine the morphological changes of CRKP treated with MDEO. CRKP was incubated in TSB at 37℃ for 18 h. Different MDEO concentrations (MIC and 2MIC) were added to the bacterial suspensions. Bacterial suspensions without MDEO served as the negative control. The bacterial suspensions were incubated at 37℃ for 0 h, 2 h, and 4 h, respectively. They were subsequently centrifuged at 5,000 × g for 5 min at 4℃. The cells obtained from the pellet were fixed in 2.5% (v/v) glutaraldehyde for 4 h and washed four times with 0.1 mol/L PBS (pH 7.4). They were successively dehydrated using 30%, 50%, 70%, 80%, 90%, and 100% ethanol for 10 min, respectively. After being dried by CO_2_ and coated with gold, the bacterial cell morphology was observed with SEM.

### Antibiofilm activity assay

CRKP cultures were grown overnight in TSB and adjusted to 0.5 McFarland. 100 µL of the culture medium was transferred into plates containing 100 µL MDEO (MIC, and 2MIC), bacterial suspensions without MDEO served as the negative control. After a 48 h incubation at 37^°^C, the biofilms were washed three times with sterile phosphate buffer saline (PBS pH 7.4) to remove free-floating planktonic bacteria. Biofilms formed by adherent organisms in the plate were stained with crystal violet (0.4% w/v). The absorbance at 570 nm was measured. Each experiment was performed independently three times. The inhibition percentage was calculated as follows:

Inhibition percentage = (OD negative control − OD treated sample/OD negative control) × 100% [15].

### Detection of CRKP cell membrane permeability

#### Protein leakage

The intracellular protein leakage of CRKP treated with MDEO (at MIC and 2MIC) for 4 h was evaluated using sodium dodecyl sulfate-polyacrylamide gel electrophoresis (SDS-PAGE) (Beyotime Biotechnology, China) [16]. Non-MDEO-treated samples were labeled as controls. After a 4 h MDEO treatment, the bacterial suspensions were centrifuged at 6,000 × g for 10 min. The sedimented cells were washed and resuspended with PBS (0.1 M, pH 7.4), then lysed using lysozymes (0.1 mg/mL) for 30 min. Subsequently, the bacterial cells were further fragmented by ultrasound treatment (power:300 W, ultrasound pulse: 4 s, interval: 5 s) for 10 min, and the intracellular soluble proteins in the supernatant were collected and preserved at -80 °C. All experimental procedures were performed on ice. The electrophoresis loading buffer (Beyotime Biotechnology, China) was mixed with 100 µl of the above-collected proteins, and SDS-PAGE was performed. The gels were stained with Coomassie brilliant blue R-250 and decolorized to obtain the isolated protein bands.

To further verify protein leakage, intracellular proteins were quantified using the BCA Protein Assay Kit (Jiancheng Bioengineering Institute, China). The same intracellular protein samples collected from the previous experiments were used. The absorption at 562 nm was measured using a 96-Well Plate Reader. Each experiment was performed independently three times.

#### Determination of AKP activity

The CRKP extracellular AKP activity after treatment with MDEO at MIC and 2MIC was assayed using the AKP kit (Jiancheng Bioengineering Institute, China) by a UV/Vis spectrophotometer (Shimadzu, China) following the manufacturer’s instructions. The samples that were not treated with MDEO served as the control group. CRKP cultures treated with different MDEO concentrations (control, MIC, and 2MIC) were centrifuged at 1,000 × g for 10 min, respectively. The supernatant was collected, and its absorbance at 520 nm was measured. Each experiment was repeated three times. One unit of AKP activity(expressed as U/g Prot) was defined as 1 mg of phenol produced by 100 ml of culture solution interacting with the matrix in a 15-minute interval [17].

### Assessment of CRKP energy metabolism

#### Determination of ATP concentration

Intracellular ATP concentrations were determined according to the method described by [18]. Control, MIC, and 2MIC treated CRKP cultures were centrifuged at 1,000 × g for 10 min, respectively. The supernatants were removed, and the cells in the pellet were suspended by adding double-distilled water. The cell suspensions were subsequently placed in a hot water bath (100 °C) for 10 min. The intracellular ATP concentrations were measured using an ATP assay kit (Jiancheng Biological Engineering Institute, China) by monitoring the absorbance at 520 nm. Every experiment was performed independently three times.

#### Determination of ATPase activity

The ATPase activity of CRKP after treatment with MDEO at MIC and 2MIC, as described above, was assayed using the ATPase assay kit (Jiancheng Bioengineering Institute, China) according to the manufacturer’s instructions. The proteins extracted from the previous experiment were used as samples. The samples absorbance was measured at 660 nm. ATPase can decompose ATP to produce ADP and inorganic phosphorus, and the inorganic phosphorus produced can be measured to determine the ATP enzyme activity. One unit of ATPase activity (expressed as U/mg prot) was defined as the amount of inorganic phosphorus produced by ATPase decomposing ATP per milligram protein per hour. Each experiment was performed independently three times.

#### Oxidative respiratory metabolism characteristics

To further determine the MDEO inhibitory effects on energy metabolism, the activity of key enzymes at irreversible reaction steps of the tricarboxylic acid (TCA) and pentose phosphate (PPP) pathways were measured. The citrate synthase activity (CS) was determined using the CS ELISA Assay Kit (Meimian Institute, China). The color change was measured by Microplate-Reader at an absorbance of 450 nm. The sample CS activity is then determined by comparing the OD_450_ of each sample to the standard curve. The isocitrate dehydrogenase (IDH), α-ketoglutarate dehydrogenase (α-KGDH), and Glucose-6-phosphate dehydrogenase (G6PDH) activities were determined using the IDH, α-KGDH and G6PDH Assay Kit (Solarbio Institute, China), respectively, by measuring the absorbance at 340 nm. One unit (U) of IGH, α-KGDH, and G6PDH enzymatic activity was defined as the production of 1 nmol of NADH per minute per 10,000 bacterial cells in the reaction system. Every experiment was performed independently three times.

### Statistical analysis

The experimental results were statistically analyzed by SPSS software (version 25.0; IBM Corp., Armonk, NY, USA). Experiments were performed in triplicate and are expressed as mean ± SD (n = 3). Analysis of variance (ANOVA) was run on all our collected data using the statistical analysis software SPSS. Multiple comparison procedures were performed with the Least-Significant Difference method if appropriate. Significant differences were determined at a significance level of *P* < 0.05. Graphs were created by GraphPad Prism software (version 8.0), (GraphPad Software, San Diego, CA 92,108, USA).

## Results

### Antibacterial activity assays

**Fig. 1:**
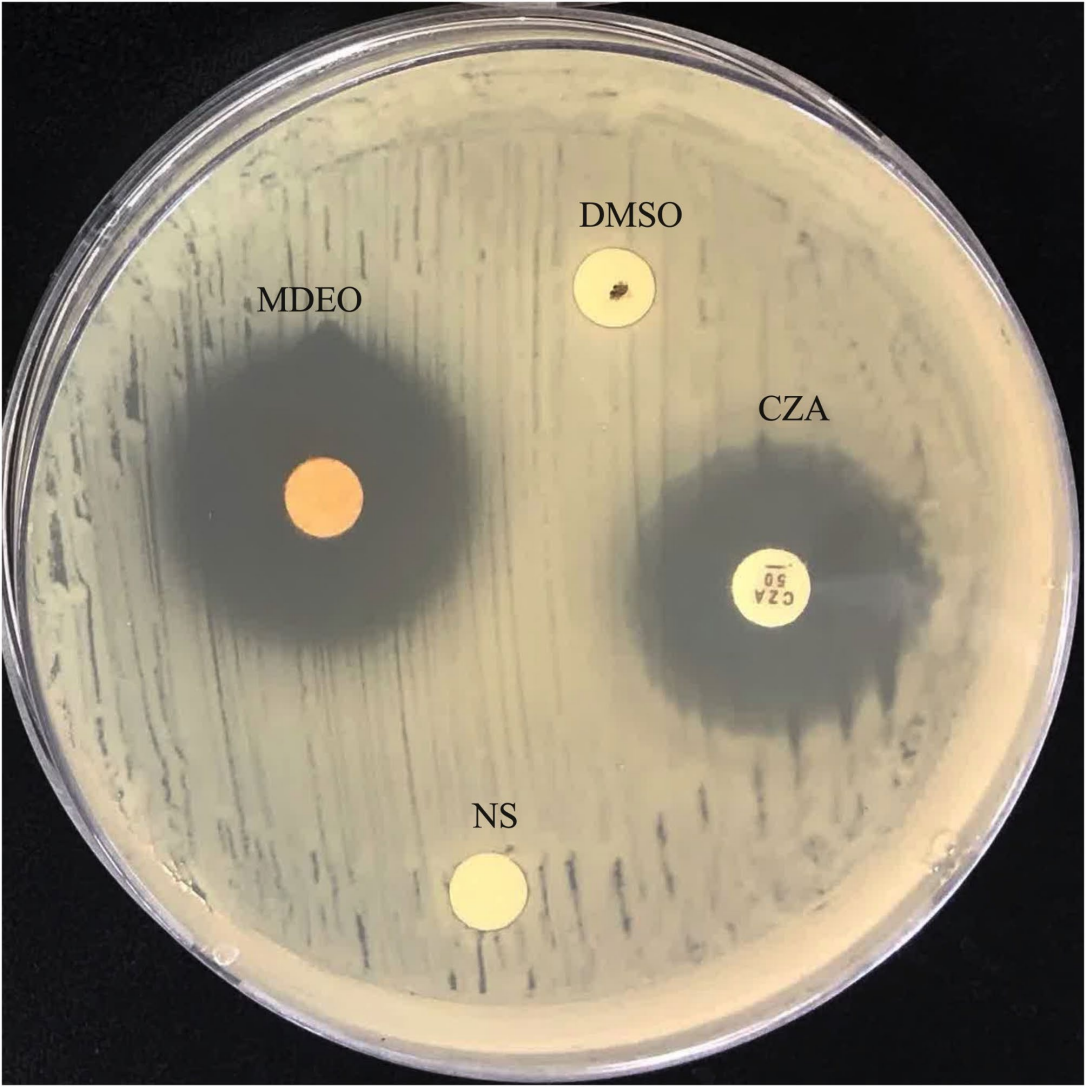
DIZ of CRKP treated by, CZA, MDEO, DMSO and NS respectively

The MDEO antibacterial activities and their specificity against CRKP were qualitatively and quantitatively assessed by measuring the diameter of the inhibition zone (DIZ), the MIC, and the MBC. The DIZ of MDEO against twenty CRKP strains was assessed, and the DIZ ranged from 29.67 ± 0.47 mm to 24.67 ± 0.47 mm (Table 1) (Fig. 1). The CRKP strain with a DIZ of 24.67 ± 0.47 mm was selected as the experimental strain for further study. The MIC and MBC of MDEO against the CRKP strains were determined by a broth microdilution assay. MIC was defined as the lowest MDEO concentration that eliminated bacterial growth after overnight incubation and was measured at 1.25 mg/ml. MBC was defined as the lowest MDEO concentration that eliminated inoculum growth when subcultured and was also determined to be 1.25 mg/ml. The equal MIC and MBC results further confirmed that MDEO is a potent bactericidal agent.

**Table 1:** DIZ of CRKP treated by, CZA, MDEO, DMSO and NS respectively

Inhibitors	Concentration (mg/mL)	Diameter (mm)
CZA	50	24.48 ± 0.63
MDEO	1000	24.67 ± 0.47
DMSO	1	0
NS	1000	0

### Growth curve analysis

**Fig. 2:**
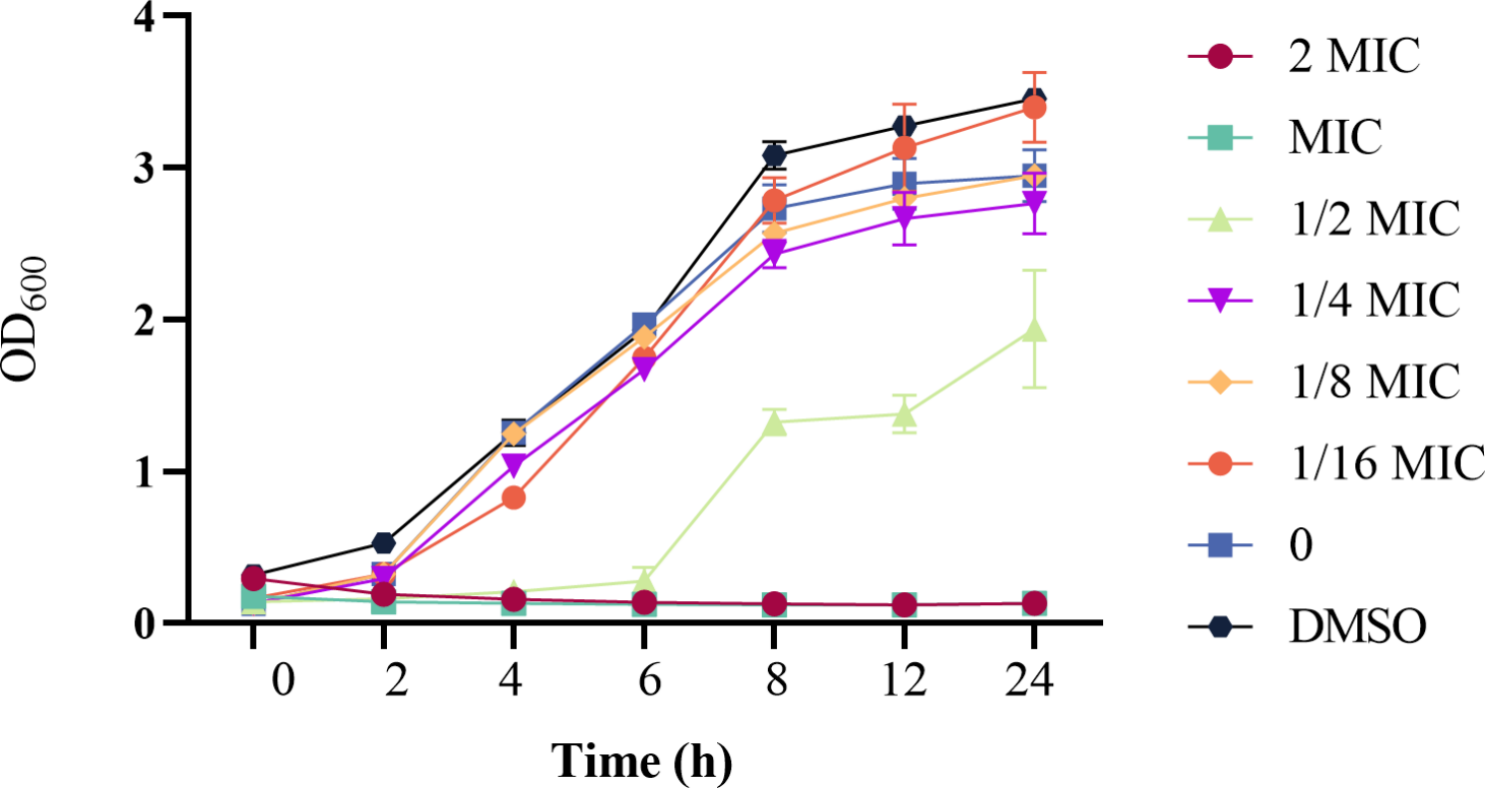
The growth curves of CRKP treatment with different concentration of MDEO. Each value represents the average of three in- dependent measurements. Bars in Fig. 2 represent the standard deviation

The bacterial growth curves can be indicative of the efficacy of MDEO against CRKP. The results are presented in Fig. 2. The CRKP growth curve in the control group rose slowly from 0 h and increased rapidly during the exponential phase from 2 to 8 h. Subsequently, it reached the stationary phase with a relatively stable optical density. At 24 h, the optical density peaked, reaching an OD_600_ value of 2.95. When treated with 1/8 and 1/4MIC MDEO, no significant difference in the growth curve was observed compared to the control group (ANOVA, n = 3, *P* < 0.05). After treatment with 1/2MIC MDEO, significant changes were observed in the growth curve compared to the control (*P* < 0.05). The lag phase was prolonged, and significantly lower growth rates and longer durations were observed in the exponential phase. At 24 h, the OD_600_ value decreased by 34% compared to the control, to 1.94. Their growth was completely suppressed when CRKP was treated with MIC and 2MIC MDEO. Therefore, MIC and 2MIC MDEO could completely inhibit CRKP growth, while 1/2MIC partially inhibited CRKP growth.

### SEM assay

**Fig. 3:**
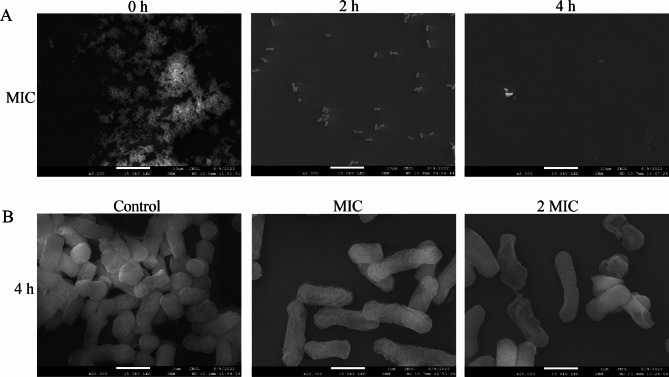
Observation of CRKP by SEM. CRKP treated with MIC MDEO for 0 h, 2 and 4 h **(A)**. CRKP treated with MDEO (Control, MIC and 2 MIC) for 4 h **(B)**

The cell density and morphological changes of CRKP treated with different MDEO concentrations were evaluated with SEM. On the one hand, the SEM images in Fig. 3A show that the CRKP number treated by MIC MDEO decreased significantly at 2 and 4 h compared to the control. CRKP not treated with MDEO were numerous, dense, and with overlapping cells. Only a few scattered cells could be observed after treatment with MIC MDEO for 2 h. Further, when treated with MIC MDEO for 4 h, there were almost no observable cells. Thus, the MDEO antimicrobial effect on CRKP is time-dependent. On the other hand, the SEM images in Fig. 3B show that the CRKP bacterial morphology after treatment with MDEO for 4 h was significantly changed compared to the untreated control. Untreated cells were rod-shaped, regular, intact, and exhibited distinct cell wall stripe features. In contrast, the MDEO-treated cells became deformed, depressed, atrophied and adhered to each other, and some of the cells were ruptured. Changes in cell morphology and disruption of membranes were more pronounced in CRKP treated with 2MIC MDEO, Compared to treatment with MIC MDEO.

### Antibacterial mechanism

#### Antibiofilm activity

**Fig. 4:**
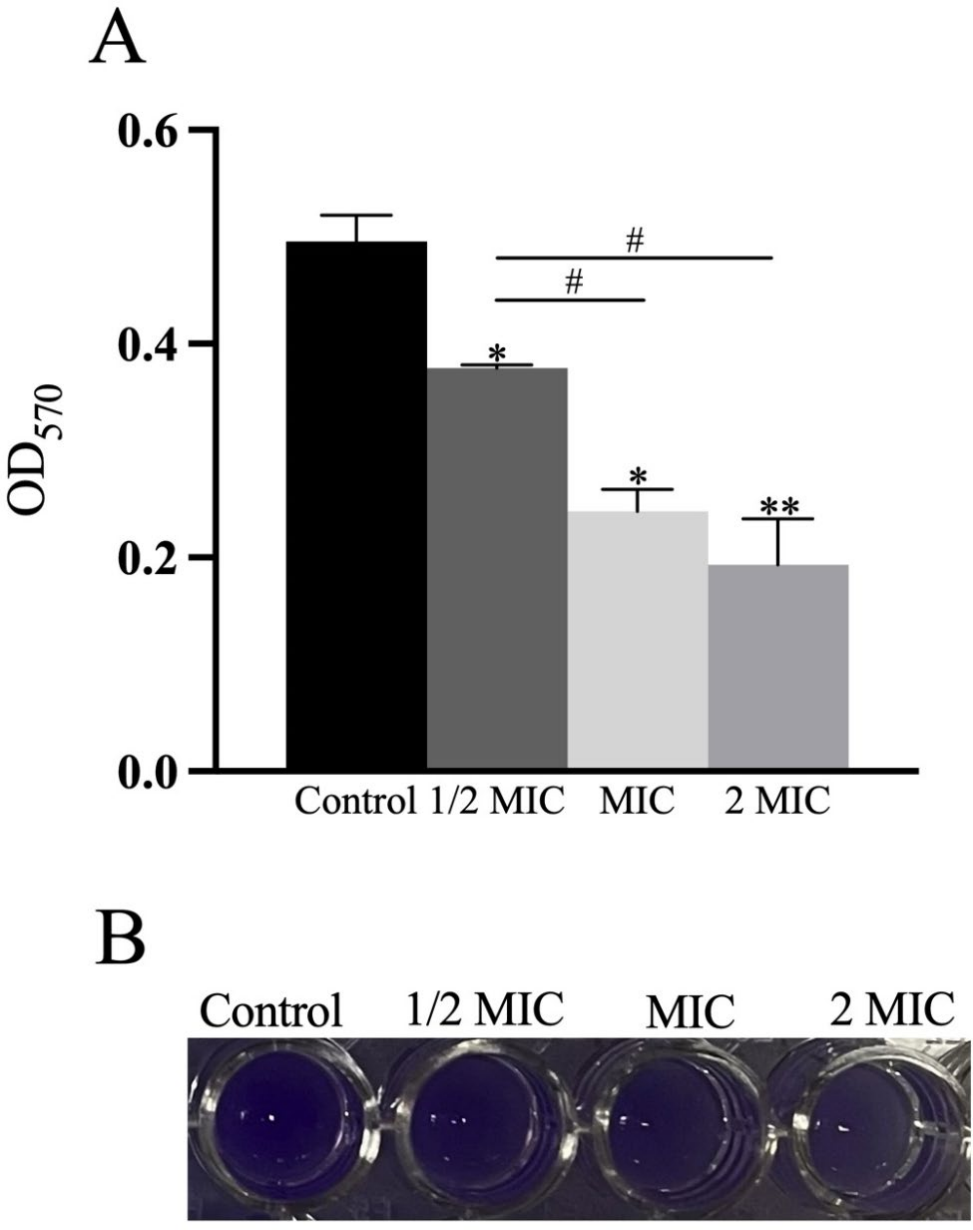
Effects of MDEO on CRKP biofilm formation. Absorbance of CRKP biofilm treated by different MDEO concentration at 570 nm **(A)**, crystal violet staining of CRKP biofilms treated with different concentrations of MDEO **(B)**. Values represent the means of triplicate measurements. Bars represent the standard deviation (n = 3). *P ≤ 0.05, #*P* ≤ 0.05, **P ≤ 0.01

A crystal violet staining assay was performed to examine the MDEO influence on CRKP biofilm formation capacity. MDEO exerted a significant inhibitory effect on CRKP biofilm formation, after crystalline violet staining, a distinct color gradient can be seen. (Fig. 4B). The biofilm absorbance of CRKP not treated with MDEO was 0.52 at 570 nm. When treated with 1/2MIC (ANOVA, n = 3, P < 0.05) of MDEO, the biofilm absorbance of CRKP was 0.38. Thus biofilm formation was inhibited by 23% compared to the control. The CRKP biofilm absorbance values at 570 nm were 0.23 and 0.19 after treatment with MDEO at MIC (ANOVA, n = 3, *P* < 0.05) and 2MIC (ANOVA, n = 3, *P* < 0.05), respectively, reduced by 51% and 60% compared to the control (Fig. 4A). The results indicated that the MDEO inhibitory effect on CRKP biofilm formation was concentration-dependent (ANOVA, n = 3, *P* < 0.05).

### Effect of MDEO on CRKP Cell membrane

#### Release of proteins

**Fig. 5:**
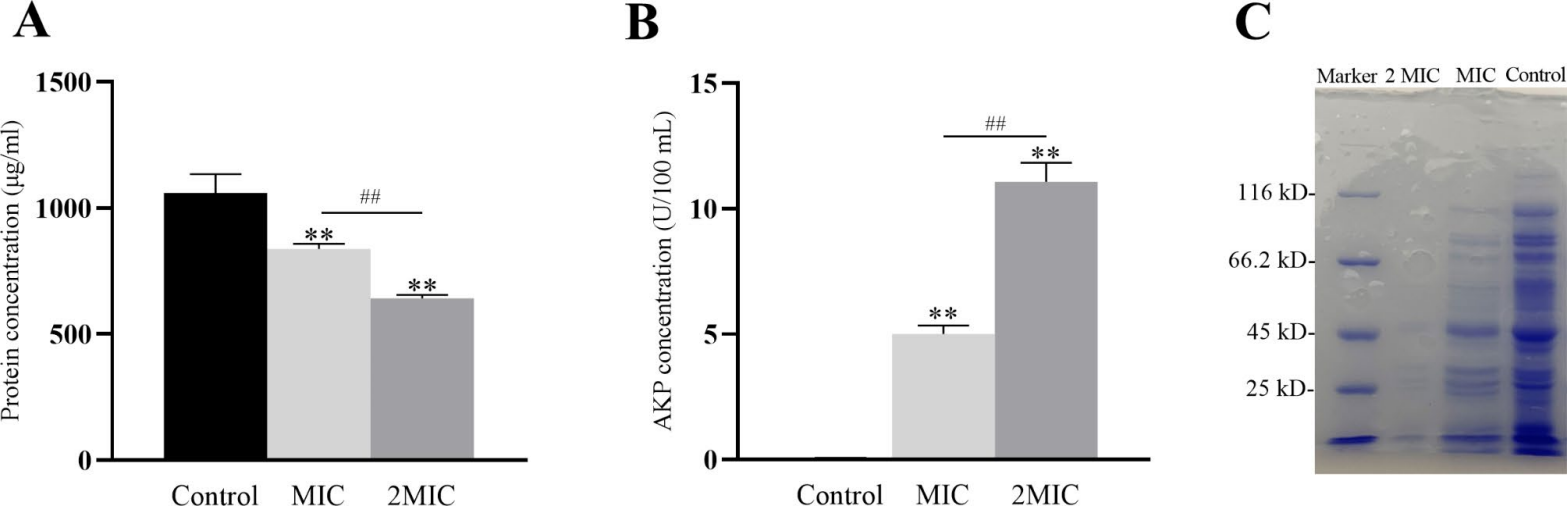
Effects of MDEO on intracellular proteins (**A** and **C**) (The full-length gels and blots are included in a Supplementary Information file) and AKP activity **(B)** of CRKP. Values represent the means of triplicate measurements. Bars represent the standard deviation (n = 3), **P ≤ 0.01, ^*##*^*P* ≤ 0.01

Proteins are macromolecules that are in their majority located intracellularly. The MDEO effects on CRKP protein leakage were analyzed by SDS-PAGE and a protein test kit. When treated with MIC MDEO, CRKP’s intracellular soluble protein bands are narrower and lighter than the control (Fig. 5C). This phenomenon became even more apparent after treatment with 2MIC MDEO, with virtually no protein bands visible compared to the control. SDS-PAGE results revealed that the intracellular soluble proteins number and type were reduced in MDEO-treated CRKP, and this effect was concentration-dependent. The protein assay kits’ results also showed the same trend (Fig. 5A). The intracellular protein concentration in the control group was 1059.9 µg/ml. In the MIC MDEO treated group, it was 838.5 µg/ml, and in the 2MIC MDEO treated group was 642.5 µg/ml. Compared to the control group, the CRKP intracellular protein treated with MIC and 2MIC decreased by 21% and 49% (ANOVA, n = 3, *P* < 0.05), respectively. Thus, the intracellular protein concentration of CRKP treated with MDEO decreased, suggesting that MDEO can damage the cell membrane, leading to the leakage of macromolecules.

#### Extracellular alkaline phosphatase (AKP) activity

AKP is a cytoplasmic enzyme that can penetrate the periplasmic space. The extracellular AKP activity was assayed by a UV/Vis spectrophotometer using an AKP kit. The extracellular AKP activity of CRKP in the non-treated control was 0.003 U/100 ml, while after treatment with MIC MDEO, it reached 5.008 U/100 ml, 1669-fold higher compared to the control. After treatment with 2MIC MDEO, the CRKP extracellular AKP activity was 11.072 U/100 ml, 3691-fold higher compared to the control (Fig. 5B). AKP is generally only released from cells with impaired cell wall permeability. Thus, MDEO can cause a rupture of the cell membrane, allowing AKP to leak out of the cell.

### Effect of MDEO on CRKP energy metabolism

#### Determination of ATP concentration

**Fig. 6:**
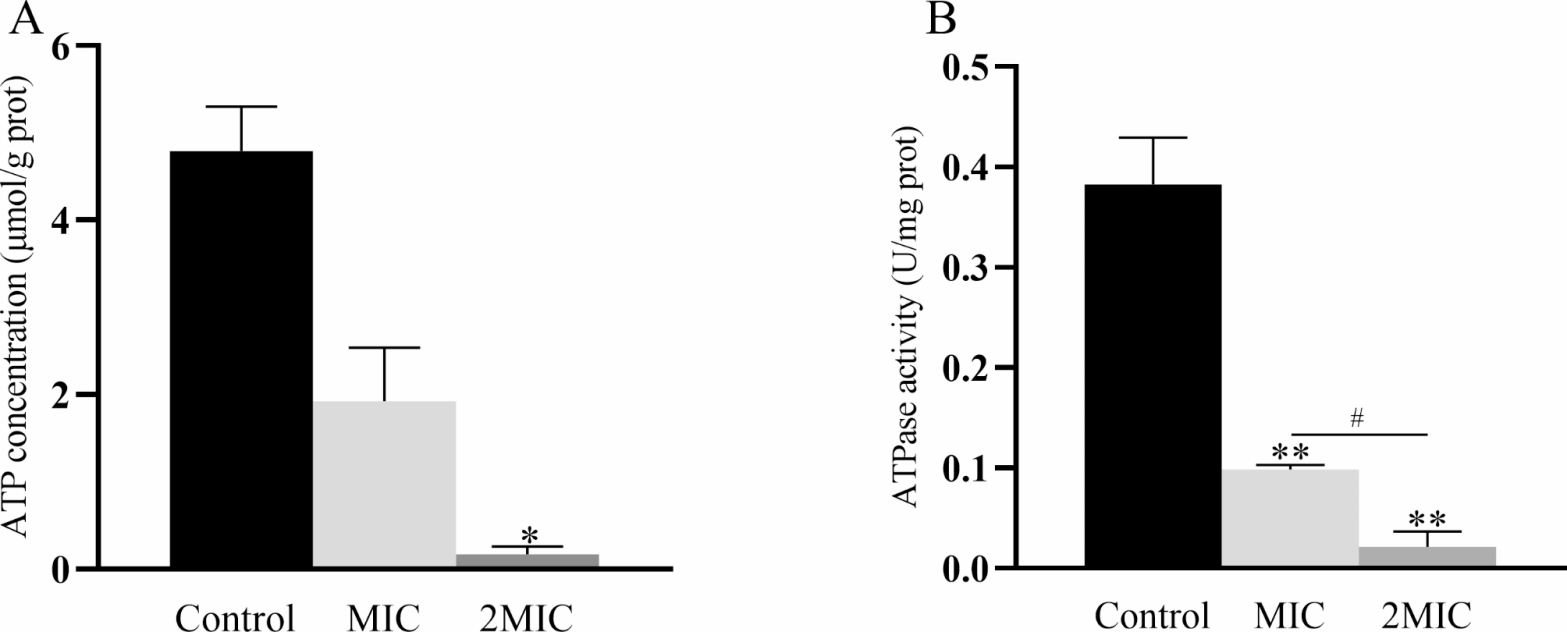
Effects of MDEO on ATP concentration **(A)** and ATPase activity **(B)** of CRKP. Values represent the means of triplicate measurements. Bars represent the standard deviation (n = 3). *P ≤ 0.05, ^*#*^*P* ≤ 0.05, **P ≤ 0.01

The ATP content in bacteria is directly related to energy metabolism, release, storage, and utilization. The effect of MDEO on CRKP intracellular ATP concentration is shown in Fig. 6A. The intracellular ATP concentration in the non-treated control was 4.22 µmol/g protein, and it decreased by 95% in CRKP treated by MIC MDEO to 0.23 U/g protein. No significant differences were observed between MIC and the 2MIC MDEO treatment groups (ANOVA, n = 3, *P *> 0.05). The intracellular ATP concentration after 2MIC MDEO treatment was 0.09 µmol/g protein, which decreased by 98% compared to the control. The results indicate that MDEO can reduce CRKP energy production, and this inhibition effect is concentration-dependent.

#### Determination of Adenosine triphosphate hydrolyzing enzyme (ATPase) activity

ATPases play key roles in various cellular functions, generating energy for solute transport and cell motility. The ATPase activity was assayed using an ATPase assay kit. The ATPase activity of CRKP in control conditions was 0.38 U/mg prot. The ATPase activity of CRKP treated with MIC MDEO decreased by 76% compared to the control, to 0.09 U/mg prot. Notably, the ATPase activity decreased with increasing EO concentration (ANOVA, n = 3, *P *< 0.05), decreasing by 95% compared to the control, to 0.02 U/mg prot, after treatment with 2MIC MDEO (Fig. 6B). Based on the above, the MDEO treatment effectively suppressed the ATPase activity of CRKP.

#### Oxidative respiratory metabolism characteristics

**Fig. 7:**
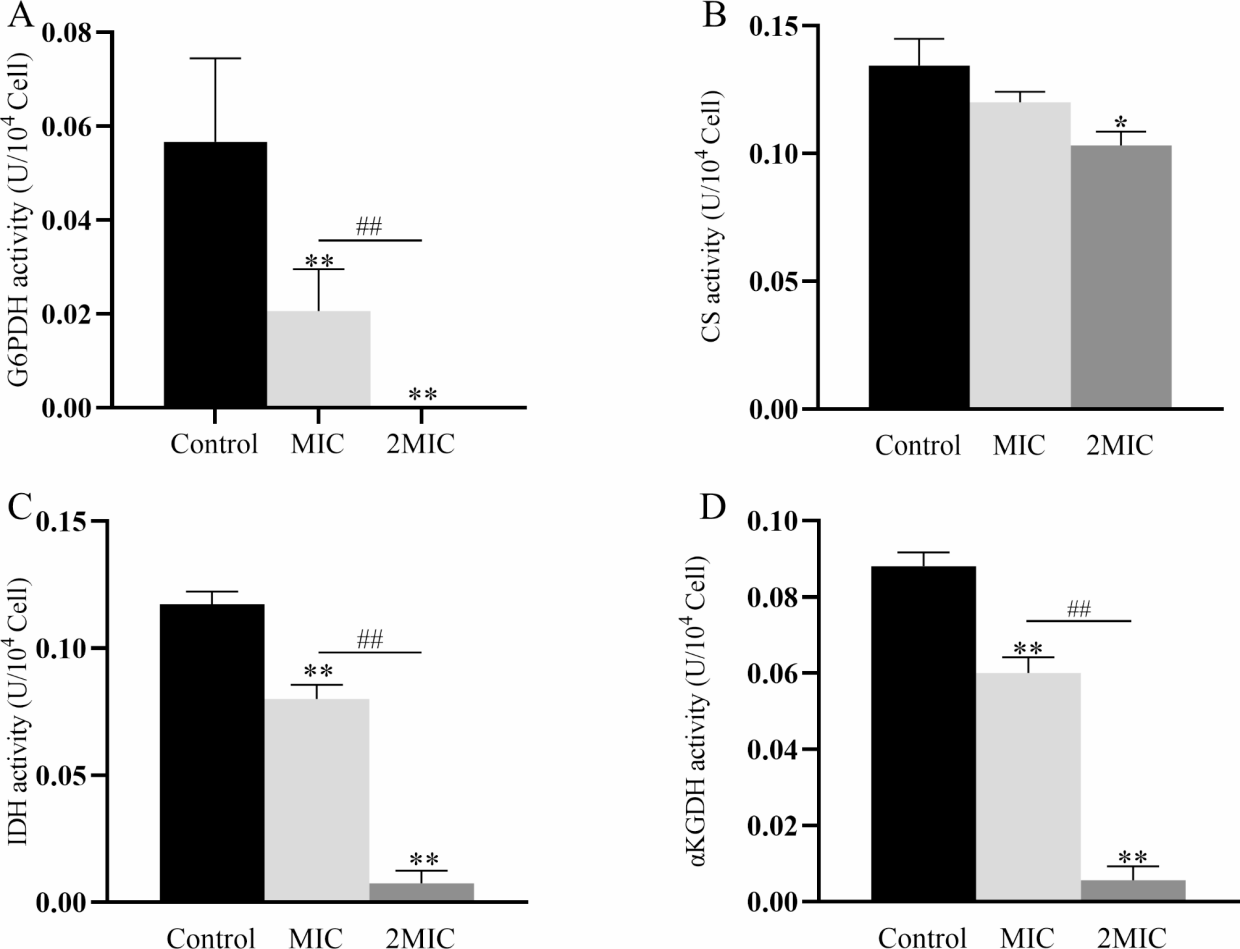
Effects of MDEO on G6PDH activity **(A)**, CS activity **(B)**, IDH activity **(C)** and α-KGDH activity **(D)** of CRKP. Values represent the means of triplicate measurements. Bars represent the standard deviation (n = 3). *P ≤ 0.05, **P ≤ 0.01, ^*##*^*P* ≤ 0.01

In the TCA pathway, the CS, IDH, and α-KGDH enzymes are key regulators of the catalytic pathway, as their reactions are irreversible. For the same reason, G6PDH is a key regulator in the PPH pathway. After treatment with MDEO at MIC and 2MIC concentrations for 4 h, the activities of all the above enzymes were lower compared to the control (Fig. 7). The activity of G6PDH (Fig. 7A), CS (Fig. 7B), IDH (Fig. 7C), and α-KGDH (Fig. 7D) in CRKP not treated with MDEO was 0.056 U/10^4^ cell, 0.134U/10^4^ cell, 0.117 U/10^4^ cells, and 0.088 U/10^4^ cells, respectively. The activity of G6PDH, CS, IDH, and α-KGDH of CRKP treated with MIC MDEO decreased by 64%, 10%, 32%, and 32% compared to the control, to 0.020 U/10^4^ cells, 0.120 U/10^4^ cells, 0.080 U/10^4^ cells, and 0.060 U/10^4^ cells, respectively. After treatment with 2MIC MDEO, the activity of G6PDH, CS, IDH, and α-KGDH of CRKP was further decreased by 100%, 22%, 94%, and 93% compared to the control, to 0 U/10^4^ cells, 0.104 U/10^4^ cells, 0.007 U/10^4^ cells, and 0.006 U/10^4^ cells, respectively. Thus, MDEO can effectively perturb oxidative respiratory metabolism by inhibiting key enzymes of the TCA and PPP pathways.

## Discussion

CRKP was first reported in 1996 [19] and quickly spread worldwide. Due to extensive antibiotics overuse, especially lactam antibiotics, the CRKP prevalence is rapidly increasing. In 2016, the United States Center for Disease Control listed Enterobacteriaceae (CRE) as an urgent threat [20]. Among CRE, CRKP is one of the key bacteria whose management is a priority. Carbapenem resistance in *K. pneumoniae* was shown to be associated with increased mortality [21, 22]. Therefore, new antimicrobial therapies are urgently needed to treat infections associated with CRKP. *Monarda didyma* is an aromatic herb of the *Lamiaceae* family, also used for food, spice, and medicinal purposes. The *Monarda* genus has a long history of medicinal use in folk medicine, known for its effects on digestive system diseases [23, 24]. In the literature, only a few studies have investigated and reported the antibacterial, antifungal, and antioxidant activities of MDEO [9, 25–27]. However, there are no published articles on MDEO antibacterial effects against multi-drug resistant (MDR) bacteria. To further expand the MDEO antibacterial spectrum and utility, we explored its antibacterial activity against CRKP strains and its potential mechanisms.

In this study, the DIZ and MIC, MBC of MDEO against CRKP indicated its significant inhibitory and bactericidal capacity. The DIZ of MDEO against CRKP was 24.67 ± 0.47 mm (Table 1) (Fig. 1), The EO antibacterial activity can be categorized into three groups: strong activity (DIZ > 20 mm), moderate activity (12 < DIZ < 20 mm), and weak activity (DIZ < 12 mm) [28]. Based on our results, MDEO exhibited strong antimicrobial activity against CRKP. Notably, the DIZ of Ceftazidime-avibactam (CZA) against CRKP was lower compared to MDEO. CZA is a third-generation antibiotic with a combination of cephalosporin ceftazidime and the novel, non-β-lactam β-lactamase inhibitor avibactam, a novel option for treating serious MDR infections [29]. These results indicate the possibility of MDEO being used as a novel antibiotic.

The MIC and MBC of MDEO against CRKP were equal (MIC = MBC = 1.25 mg/mL). An MBC/MIC ratio ≤ 4 is considered bacteriostatic, and a ratio ≥ 4 is bactericidal, respectively [30]. Therefore, MDEO has a bactericidal effect on CRKP based on this classification. In a previous study, Muntean et al. [23] found that *Mentha piperita* L. essential oil (MPEO) had a MIC value of 40 mg/mL against CRKP, equal to its MBC. Compared with MPEO, MDEO exhibits a stronger bactericidal ability. Furthermore, the MDEO antimicrobial properties were also confirmed by its effect on the CRKP growth curve (Fig. 2). MDEO at 1/2MIC could effectively inhibit the growth and reproduction of CRKP. At MIC and 2MIC, CRKP growth was completely inhibited. Moreover, changes in CRKP population numbers after treatment with MIC MDEO were observed by SEM (Fig. 3). The number of bacteria decreased progressively with increasing treatment time. SEM also clearly demonstrated that MDEO could significantly impact the normal morphology of CRKP. Cell deformation was more obvious with the increase in essential oil concentration. Obvious cell ruptures were observed in CRKP treated with 2MIC MDEO. In summary, the antibacterial capacity of MDEO against CRKP was time-dependent and concentration-dependent.

It is well known that terpenoids, alcohols, aldehydes, and esters are mainly responsible for the antimicrobial effect of essential oils. Regarding terpenoids, phenolic compounds, especially thymol, and carvacrol, possess a stronger antibacterial ability [31]. Thymol is mainly isolated from plants of the *Lamiaceae* family [31]. A previous study has shown that thymol was the most effective component of *ThymusSyriacus Boiss* essential oils against *Klebsiella pneumoniae*. This remarkable antibacterial ability of MDEO was probably due to thymol, which accounts for 69.75% of MDEO.

Biofilms, a widely observed growth pattern in which microbial communities are spatially structured and embedded in the extracellular matrix, are an important factor in *K. pneumoniae* virulence. The biofilm matrix can physically protect the bacteria while facilitating the transfer of antibiotic-resistance genes, thereby increasing microbial antibiotic resistance, bacterial durability, and proliferation [26, 32, 33]. In a previous retrospective study, CRKP, with a high capacity to produce biofilms, was significantly associated with increased mortality in infected patients [34]. Biofilm elimination requires high concentrations of antimicrobial agents, which is often impossible to realize given their associated drug toxicity [35]. EOs, as a natural product, has been extensively studied for their ability to inhibit biofilm. Eugenol [36], Paeoniflorin [37], and ursolic acid [38] presented strong inhibitory effects on CRKP biofilm formation, confirmed by FESEM and CLSM images and crystal violet staining assay. Our results suggested that MDEO displayed a significant inhibitory effect on CRKP biofilm formation (Fig. 4). When treated with MIC MDEO, the biofilm formation was reduced by 51% compared to the control. The biofilm-inhibitory effects may occur due to the inhibition of bacterial biofilm-associated gene expression. In previous studies, EOs were shown to regulate genes and proteins related to motility, Quorum Sensing (QS), and exopolysaccharides (EPS) matrix to inhibit biofilm formation [39–42].

Gram-negative bacteria are characterized by their cell envelope, consisting of an inner cytoplasmic membrane, an outer membrane, and a thin peptidoglycan cell wall interspersed between them. The outer membrane protects the bacteria from damage during infection while ensuring adequate access to the environment. Porins and secretion systems allow for secreting selected substances outside the cell. The permeability barrier provided by the cell membrane is critical for many cellular functions, such as the maintenance of the cell’s energy state, membrane-coupled energy transduction processes, solute transport, and metabolic regulation. Owing to the hydrophobic component of the outer membrane, it becomes a target for compounds contained in EO. Previous studies have shown that terpenoids of EOs can attach with the hydrophobic phenolic groups into the lipid bilayer, interacting with the polar part of the membrane, resulting in the sinking of hydrophobic benzene rings and aliphatic side chains into the inner cytoplasmic membrane. Ultimately, this changes the membrane structure, resulting in decreased elasticity, increased mobility, and increased membrane permeability. Ultimately, cell integrity is disrupted, allowing for significant cell content leakage [38, 40, 43, 44]. Therefore, the extravasation of intracellular materials is a promising predictor for assessing the cell membrane’s integrity.

AKP is located in the periplasm between the outer membrane and cell wall and is not detected in the extracellular space unless the outer membrane and cell wall have been damaged. Therefore, the detection of AKP activity in cell suspensions can reflect the integrity of the bacterial outer membrane and cell wall [45]. In a previous study, the extracellular AKP activity of *E. coli* increased after being treated with oregano essential oil (OEO) compared to the control group, indicating that the *E. coli* outer membrane had been damaged by OEO. In our study, the extracellular AKP activity of CRKP treated with MIC MDEO was 1669-fold higher compared to the control. When treated with 2MIC MDEO, it reached levels 3691-fold higher compared to the control (Fig. 5B). The higher extracellular AKP activity of bacteria was increased with MDEO concentration increase, which is analogous to the results of the growth curve and SEM of CRKP. Therefore, cell wall permeability was significantly increased, potentially due to bacterial cell wall disruption, revealing that MDEO could perturb the structure of the outer membrane and bacterial cell wall.

Moreover, the disruptive effect of MDEO on the CRKP inner cytoplasmic membrane was also confirmed by SEM and the observed intracellular protein leakage. In previous studies, eugenol, ursolic acid, and Paeoniflorin could disrupt the integrity of the CRKP cell membrane, which was confirmed by a decrease of intracellular ATP and a distinctive alteration in cell morphology [19, 36, 46]. In this study, the morphological changes of MDEO-treated CRKP were confirmed by SEM. Furthermore, as shown by the SDS-PAGE results (Fig. 5C), CRKP’s intracellular soluble protein bands were narrower and lighter when treated with MIC MDEO compared with the control group. The decrease in the amount and type of intracellular soluble proteins can explain this phenomenon. The decrease in intracellular protein concentration further confirmed the protein leakage (Fig. 5A). Intracellular proteins were reduced by 21% and 49% in CRKP treated with MIC and 2MIC MDEO, respectively, compared to the control group. Overall, MDEO can rapidly react with the outer membranes and cell walls, causing the cell membrane to lose its function. At the same time, the increase in the inner cytoplasmic membrane permeability resulted in the disruption of CRKP integrity, ultimately causing cell lysis and death.

In addition to cell membrane damage, as previously reported, metabolic disorders can also lead to bacterial death [47]. ATP is often referred to as the “molecular currency unit” of intracellular energy transfer and is the foundation for all kinds of cellular activities. In this study, CRKP intracellular ATP concentration decreased dramatically by 95% when the cells were treated with MIC MDEO compared to the control group (Fig. 6A). This significant drop in a short time suggests that ATP was leaking from the disrupted cell membranes. However, as previously reported, EOs can reduce the intracellular ATP pool by decreasing ATP synthesis and increasing hydrolysis, separately from their effects on increased membrane permeability that may lead to ATP leakage [48]. We measured key enzymes in bacterial energy metabolism to verify how MDEO affected CRKP ATP synthesis.

ATPases are enzymes involved in energy release. ATPase’s usual function is ATP production, but when freed from the driving force of the electron transport chain, it can operate in reverse and hydrolyze ATP [49, 50]. Thus, ATPase activity can measure its ability to hydrolyze ATP. ATPase plays a key role in energy production, and inhibition of its activity will significantly decrease ATP content. Finally, the ATP content reduction will lead to cell dysfunction. According to our results, MDEO inhibited ATPase in a dose-dependent manner, as the ATPase activity of CRKP decreased by 76% and 95% after treatment with MIC and 2MIC MDEO, respectively, compared to the control (Fig. 6B). This suggests that ATPase is a potential MDEO inhibitory target in CRKP. Comparable reports are consistent with our results. The activity of four MRSA ATPases decreased during LC-EO treatment [50].

TCA is the main pathway of energy production in cells. If the TCA pathway is disrupted or inhibited, microorganisms’ growth, development, and reproduction will slow down or even cease, resulting in death. The activity of key TCA pathway enzymes is an important indicator of how antimicrobial agents affect TCA. CS, IDH, and α-KGDH are rate-limiting enzymes in the TCA cycle, with important roles in regulating the TCA cycle and mitochondrial respiratory metabolism. Based on our results, the activity of three regulatory enzymes in MDEO-treated CRKP showed a downward trend in a concentration-dependent manner compared with the control group (Fig. 7B 7 C 7D). These results suggested that MDEO can effectively inhibit CRKP oxidative respiratory metabolism via TCA pathway perturbation. In a previous study, the effect of *A. villosum* Lour EO on the MRSA TCA cycle was investigated. The activities of CS, α-KGDH in the EO-treated group decreased by approximately 12.21% and 43.57%, compared to the control group, respectively. However, ICDH activity showed an increase of approximately 57.19% in the EO-treated group, which is opposite to our results [50].

PPP pathway is a metabolic pathway parallel to glycolysis. It is a major NADPH source and produces pentoses (5-carbon sugars) and ribulose-5-phosphate, a precursor of nucleic acids and other compounds. G6PDH is the key enzyme controlling the PPP pathway. In a previous study [51], the inhibitory effect of *Litsea cubeba* essential oil on MRSA respiratory metabolism mainly originated from the PPP pathway inhibition. In addition, the G6PDH activity of LC-EO-treated MRSA was significantly lower compared to the control group, verifying the inhibitory effect of LC-EO on the MRSA PPP pathway. In our study, the G6PDH activity of CRKP treated with MIC MDEO decreased by 64% (Fig. 7A). This indicates that MDEO can affect energy metabolism by perturbing the PPP pathway. Overall, MDEO can inhibit the energy metabolism of CRKP at multiple sites.

## Conclusion

Based on our results, MDEO can exert inhibitory effects on CRKP through different mechanisms. Firstly, MDEO could inhibit the biofilm formation of CRKP. Secondly, the MDEO effect on CRKP membrane structure was verified by SEM images and through qualitative and quantitative analysis of biological macromolecules and intracellular enzyme leakage after MDEO treatment. Finally, MDEO can inhibit the energy metabolism of CRKP by affecting the ATPase enzyme activity and key molecules in PPP and TCA cycle, ultimately resulting in its bacteriostatic and bactericidal effects.

### Electronic supplementary material

Below is the link to the electronic supplementary material.

Supplementary Material 1

## Data Availability

The datasets used and/or analyzed during the current study are available from the corresponding author on reasonable request.

## Abbreviations

AMR: Antibiotic resistance
MDEO: *Monarda didyma * essential oils
CRKP: Carbapenem-resistant*Klebsiella pneumoniae*
EO: essential oils
MIC: Minimum inhibitory concentration
MBC: Minimum bactericidal concentration
SEM: Scanning electron microscope
AKP: Alkaline phosphatase
ATPase: Adenosine triphosphate hydrolyzing enzyme
