# Supplementary figures and images for: In-vitro antibacterial activity and mechanism of Monarda didyma essential oils against Carbapenem-resistant Klebsiella pneumoniae
Source: BMC Microbiol. 2023 Sep 20;23:263. doi: 10.1186/s12866-023-03015-4 (PMC10512558; doi:10.1186/s12866-023-03015-4)

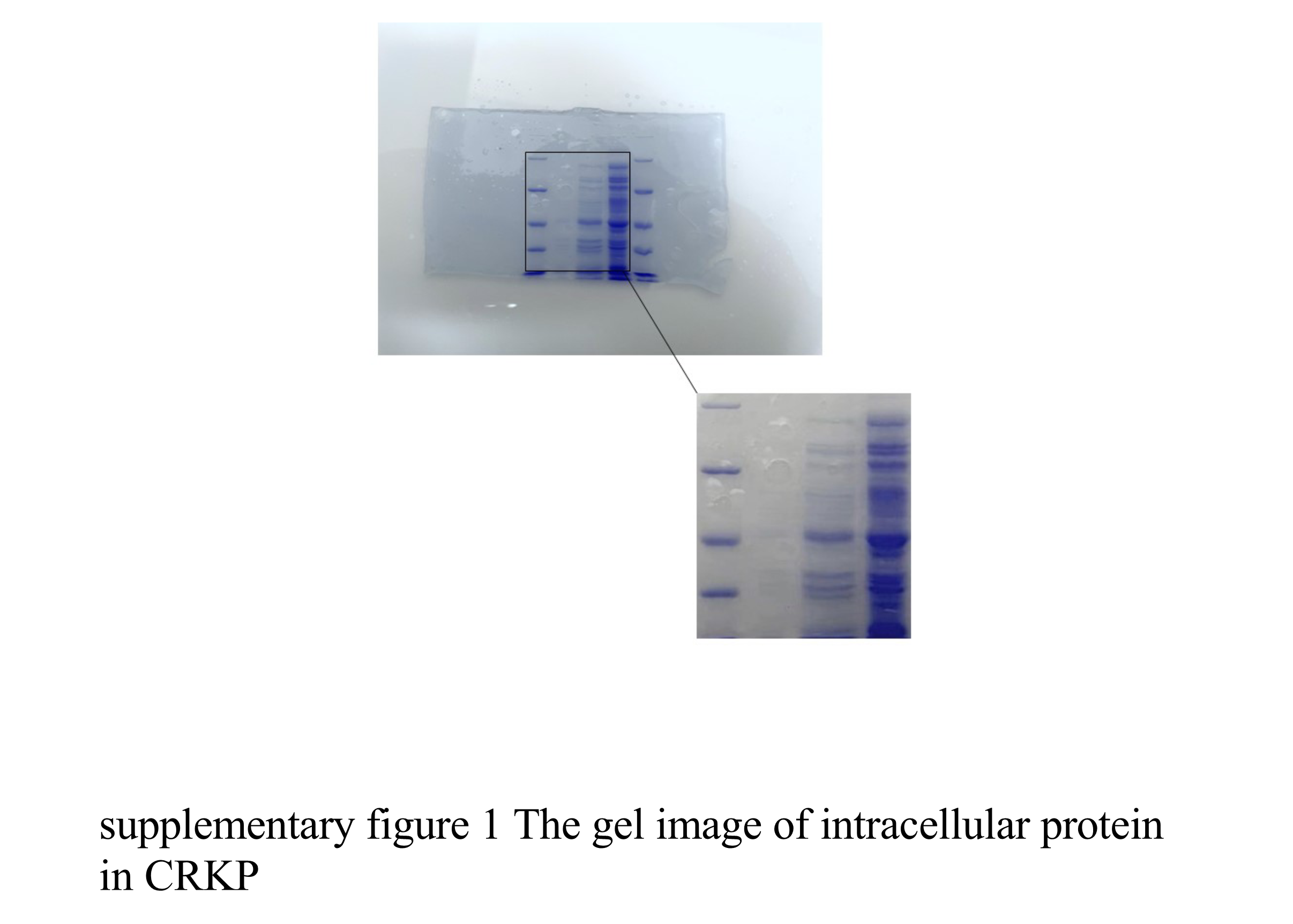

Supplement: Supplementary file 1 — Supplementary Material 1 [file 12866_2023_3015_MOESM1_ESM.tif]
